# Prevalence and species distribution of *Candida* bloodstream infection in children and adults in two teaching university hospitals in Egypt: first report of *Candida kefyr*

**DOI:** 10.1007/s15010-022-01888-7

**Published:** 2022-08-26

**Authors:** Nashwa Mohamed Reda, Reem Mostafa Hassan, Sherifa Tarek Salem, Reham Hamed A. Yousef

**Affiliations:** Clinical and Chemical Pathology Department, Faculty of Medicine, Cairo University, Cairo, Egypt

**Keywords:** *Candida albicans*, Non-albicans *Candida*, *Candidaparapsilosis*, Candidemia, Uncommon *Candida*, *Candida kefyr*

## Abstract

**Background:**

Candidemia is a pervasive problem associated with significant morbidity and mortality in health care settings. This study aimed to determine the changing distribution of *Candida* species and the emergence of uncommon species.

**Methods:**

This was a cross-sectional study performed in two Cairo University hospitals between 2019 and 2020. All *Candida* species isolates recovered from blood cultures of adults and pediatrics patients admitted to the hospitals were included. *Candida* isolates were identified by chromogenic *Candida* agar and Vitek2 YST identification card. *Candida kefyr* was confirmed by chip array.

**Results:**

*Candida* species were responsible for 1.6% of bloodstream infections in adults and 10.8% in pediatric patients. *C. albicans* was the most prevalent species representing 27.8% in adults and 48.3% in pediatrics. Non-albicans species (NAC) represented the most isolated *Candida* species among adults and pediatrics (72.2% and 51.6%, respectively) with the predominance of *C. tropicalis* (27.8% and 22.5%, respectively) followed by *C. parapsilosis* (16.7% and 10.8%, respectively). The uncommon *Candida*, which is *Candida* species other than *C. albicans*, *C. parapsilosis*, *C. tropicalis*, *C. glabrata*, and *C. krusei*, represents 16.6% and 14% of all candidemia in adults and pediatrics, respectively. Only one of each of *C. lusitaniae*, *C. utilis*, and *C. kefyr* were detected in adults. *C. lusitaniae* was the most frequently recovered uncommon *Candida* among pediatrics resulting in 6.4% of candidemia followed by *C. famata* (4.3%), *C. utilis* (2.2%), and *C. kefyr *(1.1%).

**Conclusions:**

*C. albicans* is still the primary species isolated from pediatrics and adults with candidemia despite the considerable shift to the non-albicans species. *C. tropicalis* and *C. parapsilosis* are the most prevalent NAC. The increased prevalence of uncommon *Candida* species is alarming and necessitates a prompt stewardship program.

## Introduction

*Candida* species are opportunistic fungal pathogens capable of causing a variety of infections in humans including mucosal and invasive candidiasis [1]. Candidemia, the most notable among invasive candidiasis, is a growing problem in tertiary care hospitals all over the world [2]. It is one of the most common causes of bloodstream infections(BSIs) (22%) in the United States, nevertheless bacterial BSI is much more common [3]. The global incidence of candidemia has significantly increased in the last years, which may be attributed to the widespread use of immunosuppressive therapies and broad-spectrum antibiotics. The wide use of antifungal prophylaxis also leads to infection with less susceptible *Candida* spp. [4–6]. Candidemia is associated with higher rates of morbidity and mortality in healthcare settings, especially among critically ill or immunocompromised patients or those with complicated medical conditions [7]. Although *Candida* *albicans *(*C. albicans*) is still considered a major pathogen associated with candidemia, a progressive shift from a dominance of *C. albicans* to non*-*albicans *Candida* (NAC) species has been notified by several countries [8, 9]. More than 90% of candidemia cases are attributed to one of the following five species (*C. albicans*, *C. parapsilosis*, *C. tropicalis*, *C. glabrata*, and *C. krusei*) [10, 11]. Recently, the emergence of Candidemia caused by other uncommon species such as *C. guilliermondii*, *C. lusitaniae,* and *C. kefyr* represents a new health threat to hospitalized patients [12–14]. The epidemiology and outcome of candidemia differ between pediatric and adult patients. Candidemia appears to be more frequent in neonates and young infants than in adults and is associated with a better outcome [15]. As the prevalence of *Candida* species differs between countries, regions, institutions, and different species that have variable antifungal resistance profiles it is necessary to determine the local epidemiology of candidemia for optimization of prevention and treatment [16].

This study aimed to determine the prevalence of *Candida* bloodstream infections in children and adults, the species distribution, and the emergence of rare *Candida* species from patients in two hospitals in Egypt.

## Materials and methods

### Study design

This retrospective cross-sectional study was performed in the clinical microbiology laboratories in two of Cairo University Hospitals (Kasr El-Ainy and CUSPH Cairo University Specialized Pediatric Hospital) from January 2019 to January 2020. All *Candida* species isolated from blood culture specimens from patients with septicemia were included in the study.

### Isolation of *Candida* and species identification

Nearly, 3 ml of blood collected from pediatric patients suspected of septicemia was inoculated into a pediatric blood culture bottle (BACTEC Peds Plus/F) and introduced into an automated Blood culture system BACTEC 9050 (*Becton Dickinson, USA*). For an adult patient, 5–10 ml of blood was collected in the specialized tubes for the BacT/ALERT 3D (*bioMérieux, France*) automated blood culture system. After the system was alarmed for a positive culture, Gram staining was done to confirm the presence of yeast cells. Subculture on blood agar was performed. *Candida* isolates were identified up to species level using chromogenic *Candida* agar *(Oxoid, England)* and Vitek 2 YST identification card (*bioMérieux, France*). *Candida kefyr* was first isolated in our hospital so it was confirmed by chip array.

### DNA extraction of *Candida kefyr*

DNA extraction and purification were performed using NucleoSpin Tissue mini kit (*Macherey–Nagel Gmbh &Co., Germany*), according to the manufacturer’s protocol. The DNA was stored at − 20 °C until used in PCR and chip hybridization.

### PCR amplification and hybridization

After DNA extraction, the DNA was amplified by PCR with primers supplied with the kit and hybridized onto the LCD chip. The DNA was amplified in a total volume of 25 µl using Taq polymerase (*Platinum Taq®*,* Invitrogen GmbH*,* Karlsruhe*,* Germany*), according to the instructions of the manufacturer of the LCD chip (*Fungi 2.1; Chipron GmbH, Berlin, Germany*). The cycling conditions were set to 3 min at 95 °C (initial denaturation), 30 s at 94 °C, 45 s at 56 °C, 45 s at 72 °C (35–45 repetitions for amplification), 3 min at 72 °C for final extension, and cooling at 4 °C, in a Bio-Rad Thermal Cycler PTC-200. An aliquot of 7 µL of the PCR products was run on a 2% agarose gel. In a second step, PCR product was spotted manually on LCD chips and hybridization was performed according to the instruction manual. According to the manufacturer, the array can discriminate between 25 different fungal species or species clusters, such as *C. albicans*, *C. glabrata*, *C. tropicalis*, *C. parapsilosis*, *C. krusei*, *C. dubliniensis*, *C. guilliermondii*, *C. pelliculosa*, *C. lusitaniae*, C*. lambica*, *C. kefyr*, *Aspergillus niger complex*, *Aspergillus fumigatus*, *Aspergillus flavus*, *Aspergillus terreus*, *Aspergillus nidulans*, *Mucor spp*., *Rhizomucor pusillus*, *Rhizomucor oryzae/Rhizomucor arrhizus*, *Rhizomucor azygosporus/Rhizomucor microspores*, *Rhizomucor stolonifer*, *Cryptococcus neoformans*, *Paecilomyces variotii*, *Scedosporium prolificans*, and *Lichtheimia (Absidia) corymbifera*. For scanning and final analysis, a combination of a transmission light scanner and image analysis software supplied by the manufacturer was used.

### Statistical analysis

Descriptive statistics were used to summarize the study findings. Qualitative and quantitative data values were expressed as frequency along with percentage.

## Results

During the year 2019, 3321 blood cultures from adult patients were analyzed. 34.1% (1134/3321) of blood cultures were positive for at least one organism, though only 1.6% (18/1134) of positive ones were positive for *Candida*. Among pediatric patients, 28.9% of blood cultures were positive with 10.8% (93/860) being positive for Candida species.

**Fig. 1:**
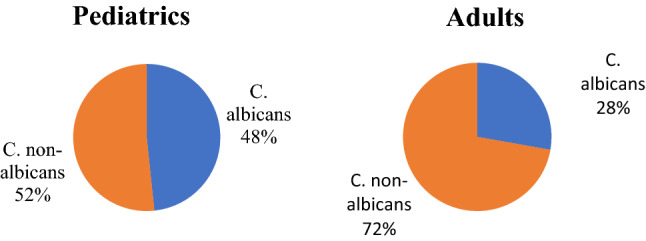
Prevalence of *C. albicans* and *non-albicans* species in pediatrics and adults

**Fig. 2:**
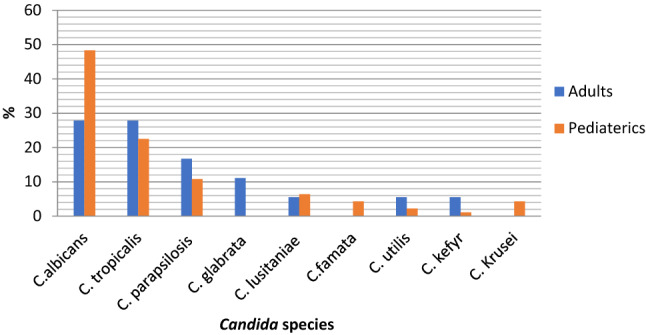
*Candida* species distribution among children and adults

Among adult patients with candidemia*, C. albicans* represented only 27.8% (5/18) while non-albicans species represented the most isolated Candida species 72.2% (13/18) (Fig. 1). *C. tropicalis* was the most common NAC causing candidemia in adults. *C. tropicalis* represented 27.8% of all candidemia cases followed by *C. parapsilosis* and *C. glabrata* (16.7, 11.1% respectively). Only one *C. lusitaniae*, *C. utilis,* and *C. kefyr* (5.5%) were detected in adults. The uncommon *Candida*, which was *Candida* species other than *C. albicans*, *C. parapsilosis*, *C. tropicalis*, *C. glabrata*, and *C. krusei*, represented 16.6% of all candidemia (Fig. 2).

While in pediatrics, there was a higher prevalence of *C. albicans* than in adults but still a predominance of non-albicans species. *C. albicans* represented 48.3% (45/93) while non-albicans 51.6% (48/93) (Fig. 1). The most common non-albicans species-causing candidemia was *C. tropicalis* (22.5%) followed by *C. parapsilosis* (10.8%), *C. lusitaniae* (6.4%), *C. krusei* (4.3%), *C. famata* (4.3%), and *C. utilis* (2.2%). One *C. kefyr (*1.1%) was also isolated from pediatric patients. The uncommon *Candida* species represented 14% of all candidemia (Fig. 2).

## Discussion

Candidemia is an alarming problem in the health care setting associated with significant mortality rates, prolonged hospital stay, and high health-related costs. The incidence of candidemia is raising globally which necessitates careful monitoring and management [17, 18]. *C. albicans*, *C. glabrata*, *C. parapsilosis*, *C. tropicalis*, and *C. krusei* are held responsible for the majority of candidemia episodes [10, 11]. In our study, we observed that *C. albicans* is still the principal species responsible for candidemia in pediatrics and adults (48.3% and 27.8%). This was in agreement with several studies showing that *C. albicans* is still the primary cause and most frequently isolated species in different countries including the United States (67%), China (36.1%), Italy (61.2%) Kuwait (32%), Saudi Arabia (38.3%) and Egypt (36%) [19–24].

Despite the high prevalence of *C. albicans* in this study, there was a predominance of the non-albicans species in both pediatric patients and adults (51.6% and 72.2%, respectively). Recently, the shift from *C. albicans* dominance to the non-albicans has been witnessed by several studies on the national and international level [24–29]. The worldwide reports of increased resistance to antifungal agents such as fluconazole and echinocandins especially with NAC together with the considerable shift in species distribution are alarming and call for prompt antifungal stewardship programs and crucial identification of *Candida* isolates to species level to allow targeted management [30].

In our study, *C. tropicalis* was the most isolated NAC from adults followed by *C. parapsilosis* and *C. glabrata*. This was consistent with a study in a tertiary care university hospital in Turkey in which *C. tropicalis* caused 14% of all candidemia cases in the general surgery department followed by *C. glabrata* (12%) and *C. parapsilosis* (8%) [31]. In parallel with our results, *C. tropicalis* was the most common species isolated from patients with solid and hematological malignancies in Taiwan (41.9%) and exhibited the highest resistance rate to azoles [32]. *C. glabrata*, in contrast to our study, was the primary NAC causing candidemia in adult patients from Israel (40%) despite the dominance of *C. tropicalis* in hematology–oncology patients [26]. Also in Denmark, Finland, Norway, and Sweden, *C. glabrata* was the foremost isolated NAC in disagreement with our results [33]. In an Egyptian study among ICU patients, *C. krusei* was the most common NAC isolated (18.5%) followed by *C. parapsilosis* (20%), *C. tropicalis* (16%*), and C. glabrata* (10%) [34].

Among pediatric patients, we observed that the most common NAC causing candidemia in this study was *C. tropicalis* (22.5%) followed by *C. parapsilosis* (10.8%). This was in agreement with a study done in India in which 84% of candidemia episodes in children were attributed to NAC with *C. tropicalis* being the foremost recovered species followed by *C. parapsilosis* [29]. In concordance with our results, some studies in Middles East regions reported the dominance of *C. tropicalis* among NAC Candidemia [35]. In Saudi Arabia at a children’s hospital at King Fahad Medical City, between January 2010 and January 2011, the most NAC included were *C. tropicalis* (23%) and *C. parapsilosis* (13.1%). *Candida famata* (5.7%), *C. lusitaniae* (4.1%) and *C. glabrata* (2.5%) [36]*.* Similar to our results, *C. tropicalis* was the 2nd most common cause of candidemia after *C. albicans* in Egyptian study during 2017 [24]*.* In contrast to our study*, C parapsilosis* was reported as the most notable NAC species among infants and children in several studies and this has been attributed to its association with catheters, which results from its capability to stick on plastic and the power to thrive in parenteral nutrition [23, 37]. In addition, in a study in the PICU of Mansoura University Children’s Hospital, Egypt, over 1 year*, C. parapsilosis* accounted for 25% of NAC candidemia followed by *C. tropicalis*, and *C. glabrata* (17% and 8%, respectively) [38]. Similar to our results, *C. glabrata* and *C. krusei* were reported infrequent causes of fungemia in pediatrics [23, 39].

The variation in species distribution could be attributed to geographical variation and certain patient-related factors as age, associated co-morbidities such as malignancies, surgery, central venous catheters insertion [40]. In addition, the types of antifungals empirically used in the region may influence the distribution of species. It was documented that the use of fluconazole promotes *C. glabrata* and *C. krusei* infections while caspofungin usage to a lesser extent promotes infection by *C. parapsilosis*, *C. glabrata*, and *C. krusei* [41].

Seldom seen *Candida* species isolated from blood are increasingly reported in the literature in the last years [12, 42–44]. This is presumably due to the advances in mycological identification methods allowing identification of isolates to species level. However, the possibility of increased emergence of previously “nonpathogenic” species as true opportunistic pathogens should be considered especially with rising numbers of immunocompromised individuals.

In our study, the uncommon *Candida* species resulted in 14% and 16.6% of candidemia cases in pediatrics and adults, respectively. We reported a higher incidence of uncommon candidemia than in several studies. In general, the documented rates of uncommon *Candida* resulting in BSIs is less than 10% [13, 45–47]. Higher frequencies are observed also among cancer patients in MD Anderson Cancer Center hospital and a pediatric medical center in Taiwan (12% and 14%, respectively) [48].

In pediatrics, *C. lusitaniae* was the most frequently recovered uncommon Candida species followed by *C. famata and C. utilis*. In contrast to our results, *C. guilliermondii* has been the most frequently isolated uncommon *Candida* species among pediatric patients in several studies [12, 43, 49].

*C. lusitaniae* is an opportunistic yeast pathogen that is often mentioned by the capability to develop resistance to amphotericin B during treatment and may manifest as breakthrough infection in immunocompromised patients on amphotericin B therapy [50]. In line with our results, *C*. *lusitaniae* was recovered from 4% of the pediatric patients in a multicenter observational study in the United States from 2007 to 2011 [51]. A slightly lower incidence of *C*. *lusitaniae* candidemia was demonstrated in Kuwait. *C*. *lusitaniae* was detected at a higher frequency among infants less than 1 year resulting in 14.6% of invasive infections in Prospective Antifungal Therapy registry (PATH) data [44]. Also, *C. lusitaniae* was the second most common cause and accounted for 18.8% of candidemia caused by uncommon *Candida* species in a medical center in Taiwan [12].

Among adults, *C. lusitaniae*, *C. utilis,* and *C. kefyr* were recovered at a low frequency and each of them constituted 5.5% of candidemia cases. In contrast to our study, C*. guilliermondii* (41%) was the most commonly recovered from cancer patients in a retrospective study over 16 years period in Texas followed by *C. lusitaniae* (28%), *C. kefyr* (19%), *C. famata* (10%), and *C. dublinensis* (1%) [13]. On the other hand in a study at a university hospital in China analyzing candidemia among cancer patients found that *C. lusitaniae* and *C. famata* were held responsible for 3.8% of candidemia [45]. Limited data is available in the literature about the prevalence and species distribution of uncommon *Candida* in Egypt.

*C. kefyr* is a rare emerging *Candida* species that can cause candidemia especially in patients with hematologic malignancies [52]. It inhabits the gastrointestinal tract and is associated with the consumption of dairy products harboring this species [53]. In our study, *C. kefyr* was reported for the first time in our hospitals with a very rare frequency. Only one isolate was detected in each of the adult and pediatric groups (5.5% and 1.1%, respectively). The rate of isolation of *C. kefyr* ranges from 0.4% to 22.2% in different countries. The highest incidence (22.2%) was demonstrated in a surveillance study in France over 12-year periods [54]. On the other hand, the frequency of *C. kefyr* isolation from blood specimens was only as low as 0.4% in a 10-year study in Canada [55]. In a children's hospital in India, four *C. kefyr* (9%) were recovered from the blood of patients with candidemia [29]. Although *C. kefyr* is thought to have a high susceptibility to all antifungal drugs, recent studies reported acquiring resistance to echinocandins during treatment [56, 57].

Our study has several limitations that to be considered. First, the limited number of candidemia caused by individual uncommon *Candida* species. Second, we lack the information about the clinical history, underlying risk factors, and prior use of antibiotics or antifungals that could help to clarify the causes of shifting to non-albicans candidemia and the emergence of rare species. Third, we did not perform an antifungal susceptibility profile that is very important to assess the resistance pattern of NAC and infrequent Candida species and establish a policy for institutional empirical antifungal usage.

In conclusion, *Candida* species is an alarming pathogen causing BSIs in pediatrics and adults. Although *C. albicans* is still the primary species isolated from pediatrics and adults with candidemia, a notable shift to NAC candidemia especially by *C. tropicalis* and *C. parapsilosis* was observed in our study. We noted a higher incidence of candidemia attributable to uncommon *Candida* which necessitate analyzing the risk factors associated with this emergence and studying the antifungal susceptibility pattern of these species. *C. kefyr* was isolated for the first time in our hospital. The increased resistance associated with NAC and the correlation between the empirical uses of antifungals and the emergence of uncommon *Candida* species reported in the literature prioritizes the antifungal stewardship program and targeted management.
